# Ketosis-Onset Diabetes and Ketosis-Prone Diabetes: Same or Not?

**DOI:** 10.1155/2013/821403

**Published:** 2013-04-23

**Authors:** Beiyan Liu, Changhua Yu, Qiang Li, Lin Li

**Affiliations:** Endocrinology and Metabolism Department of the Second Hospital Affiliated to Harbin Medical University, Harbin Medical University, Harbin, Heilongjiang Province 150086, China

## Abstract

*Objective*. To compare clinical characteristics, immunological markers, and **β**-cell functions of 4 subgroups (“A**β**” classification system) of ketosis-onset diabetes and ketosis prone diabetes patients without known diabetes, presenting with ketosis or diabetic ketoacidosis (DKA) and admitted to our department from March 2011 to December 2011 in China, with 50 healthy persons as control group. *Results*. **β**-cell functional reserve was preserved in 63.52% of patients. In almost each subgroup (except A−  **β**− subgroup of ketosis prone group), male patients were more than female ones. The age of the majority of patients in ketosis prone group was older than that of ketosis-onset group, except A−  **β**− subgroup of ketosis prone group. The durations from the patient first time ketosis or DKA onset to admitting to the hospital have significant difference, which were much longer for the ketosis prone group except the A+ **β**+ subgroup. BMI has no significant difference among subgroups. FPG of ketosis prone group was lower than that of A−  **β**+ subgroup and A+ **β**+ subgroup in ketosis-onset group. A−  **β**− subgroup and A+ **β**+ subgroup of ketosis prone group have lower HbA1c than ketosis-onset group. *Conclusions*. Ketosis-onset diabetes and ketosis prone diabetes do not absolutely have the same clinical characteristics. Each subgroup shows different specialty.

## 1. Introduction

 Atypical or ketosis-prone type 2 diabetes was first reported by Winter et al. in 1987 in black Americans [1] and is distinguishable from other subtypes of diabetes by clinical, immunological, and biological features. Till now, there are two kinds of phrases describe this type of disease: ketosis-prone diabetes [2–4] and ketosis-onset diabetes [5, 6]. One objective for classification of a disease is the opportunity to study its epidemiology, etiology, and pathogenesis to provide various effective interventions for its prevention and treatment. What is the connection between ketosis-onset diabetes and ketosis-prone diabetes? Do they mean the same kind of diseases? Or are they just different names that change as time passing by? Ketosis-prone diabetes (KPD) is defined as a widespread, emerging, heterogeneous syndrome characterized by patients who present with DKA or unprovoked ketosis but do not necessarily have the typical phenotype of autoimmune type 1 diabetes [7, 8]. While ketosis-onset diabetes patients present with ketosis or ketoacidosis without known diabetes [9, 10], some investigators defined ketosis-onset diabetes as diabetes with the presence of diabetic ketosis and in the absence of glutamic acid decarboxylase (GAD) and tyrosin phosphatase (IA-2) autoantibodies. We choose patients separately fit for each of the above conditions to study. We mean to find if patients described by the two phases have the same range and feature. The aim of our test and comparison is to find a more accurate classification and denomination, which help the clinicians to make therapeutic regimen and judge the prognosis.

Maldonado et al. 2003 [2] evaluated patients with diabetic ketoacidosis for *β*-cell autoimmunity and human leukocyte antigen (HLA) class II alleles, with longitudinal measurements of *β*-cell function and biochemical and clinical parameters. They were classified into four A*β* groups, based on the presence of glutamic acid decarboxylase (GAD) 65, GAD 67, or IA-2 autoantibodies (A+ or A−) and *β*-cell functional reserve (*β*+ or *β*−). The group distribution was A+  *β*−, A−  *β*+, A−  *β*−, and A+  *β*+. This “A*β*” classification was cited in this study.

## 2. Method

All northeast Chinese patients with newly diagnosed diabetes presenting with an acute-onset ketosis admitted to our department between March 2011 and December 2011. There were 3 groups in our study: ketosis-onset group, ketosis-prone group, and healthy control group. Ketosis-onset diabetes was defined as follows: (1) patients were admitted to the hospital with ketosis (or DKA as the first symptom), (2) newly onset diabetes, diagnosed diabetes by clinical symptoms and the laboratory test after admission [11, 12]. Ketosis-prone diabetes was defined as follows: (1) newly occurred diabetes, having typical polydipsia, polyuria, polyphagia, and extenuation within 6 months; (2) ketosis (urine ketone body above 2+) or ketoacidosis no more than 6 months after onset. Both of the two groups should match the condition that (3) there are no obvious incentives such as infection, surgery, and trauma; (4) pregnancy diabetes, drug and pancreatic exocrinosity diseases, and endocrine diseases caused secondary diabetes were excluded [13–15]. A total of 159 consecutive, unrelated Chinese patients were investigated. The data included clinical characteristics, immunological markers, and *β*-cell function. During the study, recombinant human regular insulin was given by continuous intravenous injection, until the urinalysis is negative for 3 continuous days. The insulin therapy was stopped 1 h before study measurements. The target plasma glucose level during the infusion was 4.4–5.6 mmol/L. Participants received a weight-maintaining diet of 30 kcal/kg/24 h, during their hospitalization.

Data were collected on clinical characteristics (age, gender, symptoms, family history of diabetes, anthropometric features: height, weight, and so forth), biological parameters (fasting plasma glucose (FPG), urine ketone body (above 2+), total and high-density lipoprotein (HDL) cholesterol, and triglycerides were performed once at the time of the study in a fasting state), glycosylated hemoglobin (HbA1c), immunological markers, and *β*-cell function. HbA1c was detected by HLC-723G7 automatic HbA1c analyzers (Japan Tosoh Corporation). Glutamic acid decarboxylase (GADA) and tyrosin phosphatase antibodies (IA-2A) were detected by radioimmunoprecipitation (Euroimmun, Germany). Serum was analyzed for the presence of glutamic acid decarboxylase (GAD) 65 and IA-2 autoantibodies by highly sensitive and specific quantitative radioligand binding assay methods, as reported previously [16, 17], with recent modifications [18].

One of the autoimmune antibodies positive considered as A+, either antibody negative considered as A−. *β*-cell function was assessed at least 1 week after the acute episode, when patients were in a nearly normoglycaemic state. C-peptide levels were assessed at fasting state and 6 minutes after intravenous administration of 1 mg glucagons by the radioimmunological method using a commercial kit (Immunotech, France). *β*-cell functional reserve was considered preserved if the fasting C-peptide level was >0.56 *μ*g/L.

According to test results of, respectively, 79 and 80 cases, patients were divided into 4 subgroups: group 1 as A+ *β*−, group 2 as A− *β*−, group 3 as A− *β*+, group 4 as A+ *β*+.

Statistical analysis was performed using SPSS 11.5 statistical software. Results are expressed as mean and standard deviation (SD), nonnormal data using nonparametric analysis. Comparison between the two groups was made using *t*-test, multiple comparison using analysis of variance between groups. 

## 3. Result

A total of 159 patients (64 women) aged from 7 to 72 years presenting with ketosis or DKA were investigated. Among them, 6 (3.77%) reported a family history of diabetes. 45.9% patients complained of polyuria and polydipsia, with a median duration of 76 days (range: 1 day to 6 months), and 32.7% reported weight loss. BMI was ≥25 kg/m^2^ in 27% of cases. GADA were detected in 31 (19.5%) and IA-2A were positive in 23 (14.5%) of patients. At least one immunological marker was detected in 33 cases (20.8%). *β*-cell functional reserve was preserved in 63.52% of patients.

According to immunological markers (present A+ or absent A–) and *β*-cell functional reserve (present *β*+ or absent *β*–), patients of the two groups were divided into 4 subgroups: ketosis-onset diabetes group: 14 patients (17.72%) were A+ *β*−, 14 (17.72%) were A− *β*−, 40 (50.63%) were A−  *β*+, and 11 (13,92%) were A+ *β*+; ketosis-prone diabetes group: 20 patients (25%) were A+ *β*−, 10 (12.5%) were A− *β*−, 41 (51.25%) were A−  *β*+, and 9 (11.25%) were A+ *β*+.

The data of each subgroup was compared, respectively, to the subgroup of another group. In almost each subgroup (except A− *β*− subgroup of ketosis-prone group), male patients were more than female ones. The age of the majority of patients in ketosis-prone group was older than that of ketosis-onset group, except A− *β*− subgroup of ketosis-prone group. The onset of ketosis-prone diabetes group is much later than that of ketosis-onset diabetes group. All the patients received intravenous insulin treatment until urinalysis showed negative for 3 continuous days. The duration means the time from the first ketosis or ketoacidosis onset to the patient admitting to the hospital. The durations have significant difference, which were much longer for the ketosis-prone group except for the A+ *β*+ subgroup. BMI has no significant difference among each subgroup. FPG of ketosis-prone group was lower than that of A−  *β*+ subgroup and A+ *β*+ subgroup in ketosis-onset group. A− *β*− subgroup and A+ *β*+ subgroup of ketosis-prone group have lower HbA1c than ketosis-onset group (Tables 1, 2, and 3). Triglycerides have significant difference in *β*+ subgroups. The A+ *β*+ subgroups in both groups are older and more like patients with Latent Adult onset autoimmune diabetes cases. Comparing to healthy control group, both ketosis-onset and ketosis-prone groups have higher FPG, HbA1c, and triglycerides (Table 4).

## 4. Discussion

In recent decades, there is a form of diabetes identified because of ketosis or DKA as the first symptom, which does not necessarily fit the typical characteristics of autoimmune type 1 diabetes mellitus. Because the etiology of this form of diabetes is unclear and the classic markers of autoimmune destruction of islet beta cells are also absent, the World Health Organization and the American Diabetes Association have classified this subtype as “idiopathic type 1 diabetes” or “type 1B” [19]. In present articles, there are main two phrases to name this form of disease: ketosis-onset diabetes and ketosis-prone diabetes. The result showed that ketosis-onset diabetes patients and ketosis-prone diabetes ones do not have absolutely the same clinical characters, and each subgroup showed different specialty. A−  *β*+ subgroup composes the main part of each group (both over 50 percent). There is ambiguously some kind of trend that ketosis-prone group had a later onset and milder clinical manifestations compared to ketosis-onset diabetes group. It seems that ketosis-onset group is more like type 1 diabetes while ketosis-prone group is more close to type 2 diabetes. The growing evidence, including older age at onset, higher rates of obesity, and genetic predisposition among affected individuals, suggests that ketosis-prone diabetes should be considered as a subset of type 2 diabetes or ketosis-prone type 2 diabetes [20, 21].

Although the experimental indexes in this study are not complete, but they are representatively enough to illustrate the problem. Physicians have kept trying to find a more scientific and accurate way to classify diabetes to understand mechanism and prognosis of this kind disease more better.

Ketosis-prone diabetes (KPD) is a widespread, emerging, heterogeneous syndrome characterized by patients who present with diabetic ketoacidosis or unprovoked ketosis but do not necessarily have the typical phenotype of autoimmune type 1 diabetes [22–24]. Multiple, severe forms of *β*-cell dysfunction appear to underlie the pathophysiology of KPD. The resulting “A*β*” classification system of KPD has proven to be highly accurate and predictive of such clinically important outcomes as glycemic control and insulin dependence, as well as being an aid to biochemical and molecular investigations into novel causes of *β*-cell dysfunction [25, 26]. These results demonstrate that patients with ketosis-onset diabetes are a heterogeneous group in which type 1 diabetes maybe more frequent cause. Ketosis-prone diabetes with remission is a well-known subtype of type 2 diabetes rather than type 1 [27, 28]. The acute presentation at diagnosis or sometimes later is explained by a functional and partially reversible *β*-cell deficiency [29]. There were no significant group differences in the BMI, which illustrated that the patients' posture of the two groups showed nearly the same feature. The phrase “ketosis-onset diabetes” is used more often in Japanese and Chinese articles, while “ketosis-prone diabetes” is used more frequently in other countries' articles [30]. The difference may be caused by racial differences. Recently, the term “ketosis-prone type 2 diabetes” has entered the literature. In general, this term refers to the A−  *β*+ KPD subgroup or, in some instances, is even further restricted to those A−  *β*+ patients who present with “unprovoked” DKA or ketosis and new onset diabetes [31].

A− *β*+ patients comprise the largest subgroup of KPD patients and are the ones who most commonly come to the notice of physicians because they present with DKA and yet have all the clinical features and subsequent behavior of type 2 diabetes; hence, ketosis-prone type 2 diabetes is certainly a fitting description for them. A+ *β*− KPD is synonymous with classic, early onset autoimmune type 1 diabetes; A+ *β*+ KPD may overlap with LADA. However, there are differences between LADA, as recently defined by the Immunology of Diabetes Society, and A+ *β*+ KPD patients; most importantly, the definition of LADA excludes patients who require insulin within the first 6 months after diagnosis, whereas the majority (90%) of A+ *β*+ KPD patients present with DKA as the first manifestation of diabetes and therefore require insulin at the start [32, 33].

Extensive HLA typing has found that the frequencies of major class II alleles associated with susceptibility to autoimmune type 1 diabetes are not significantly higher in A−  *β*− KPD patients than in ethnic matched population controls, whereas they are significantly higher in A+ *β*− KPD patients [34–36].

In summary, the classification of diabetes using insulin secretion evaluation and immunological markers, which seems to be the most effective scheme, allowed us to distinguish 4 subgroups of patients. In more than half of the cases (autoimmune type 1 and ketosis-prone type 2 diabetes), patients may be diagnosed by classical characteristics [37]. However, the other subgroups may require further investigations, such as repeat testing of *β*-cell function or genetic studies.

We suggest a coincident name to address this kind of diabetes which means they may have the same clinical characteristics, biochemical parameters, prognosis, mechanism, and so forth. Maybe we can even classify diabetes into ketosis-onset diabetes and non-ketosis-onset diabetes; regardless of the causes of diabetes, the onset displays ketosis or DKA or neither of them. If we keep on exploring the mechanism, perhaps we will find that there are only two prognoses of this form of diabetes: insulin-depending and non-insulin-depending diabetes which may be related to the initial onset.

Someone suggest that the four groups can be considered as type 1 DM, idiopathic type 1 DM, latent autoimmune diabetes in adults (LADA), and type 2 DM, respectively, according to their clinical characteristics, biochemical parameters, and therapeutic consequences. We consider that maybe we will find that only few cases are idiopathic, without motivation, as the study is undertaken more deeply. Most of these kinds of diabetes can be classified in to typical groups such as type 1 diabetes. We propose that the phrase “ketosis-onset diabetes” to may be used to describe the patients onset with unprovoked ketosis or DKA, and the phrase “ketosis-prone diabetes” to call those with causes. In this way, the readers could understand it better, whether the symptoms have reasons or it is a new case. Our research on race and the number of cases may have certain limitations. We will do further study in the future to search for the ignored causes of ketosis or DKA and the nature and mechanism of this of form diabetes.

## Figures and Tables

**Table 1:** Ketosis-onset diabetes clinical feature.

Group	A+ *β*−	A− *β*−	A− *β*+	A+ *β*+
	(*n* = 14)	(*n* = 14)	(*n* = 40)	(*n* = 11)
Gender (male : female)	4 : 3	11 : 3	3 : 2	8 : 3
Age (year)	23 ± 8.43	38.5 ± 13.43	45.73 ± 8.15	38.64 ± 11.24
Age range (year)	7–31	25–50	37–55	27–51
Duration (days)	29.64 ± 22.26	39.79 ± 35.27	60.2 ± 58.38	57.91 ± 51.36
BMI (kg/m^2^)	20.21 ± 2.16	21.91 ± 3.95	24.19 ± 2.67	22.73 ± 2.53
FPG (mmol/L)	16.85 ± 4.89	17.83 ± 4.57	14.13 ± 2.86	18.92 ± 7.10
HbA1c (%)	13.44 ± 2.89	12.89 ± 2.59	11.08 ± 2	12.45 ± 2.92
Triglycerides (mmol/L)	2.70 ± 2.23	2.74 ± 2.03	2.87 ± 1.73	2.63 ± 1.94

**Table 2:** Ketosis prone diabetes clinical feature.

Group	A+ *β*−	A− *β*−	A− *β*+	A+ *β*+
	(*n* = 20)	(*n* = 10)	(*n* = 41)	(*n* = 9)
Gender (male : female)	11 : 9	2 : 3	22 : 19	7 : 2
Age (year)	33.85 ± 11.24	35.7 ± 12.44	50.12 ± 9.34	43.44 ± 8.59
Age range (year)	22–45	23–48	40–72	35–52
Duration (days)	95.8 ± 84.9	101.9 ± 78.7	100.39 ± 81.68	112.11 ± 75.88
BMI (kg/m^2^)	21.07 ± 2.37	23.23 ± 3.08	23.49 ± 2.71	23.14 ± 3.31
FPG (mmol/L)	18.57 ± 5.92	16.57 ± 3.65	12.26 ± 2.96	11.76 ± 2.6
HbA1c (%)	12.46 ± 2.93	10.87 ± 2.0	10.34 ± 2.0	9.64 ± 1.17
Triglycerides (mmol/L)	2.82 ± 2.53	2.78 ± 1.76	3.13 ± 1.98	2.91 ± 2.04

**Table 3:** Comparison between ketosis-onset and ketosis prone groups (*P* value).

Group	A+ *β*−	A− *β*−	A− *β*+	A+ *β*+
Gender (male : female)	NS	NS	*P* < 0.05	NS
Age	*P* < 0.05	NS	*P* < 0.05	NS
Duration	*P* < 0.05	*P* < 0.05	*P* < 0.05	NS
BMI	NS	NS	NS	NS
FPG	NS	NS	*P* < 0.05	*P* < 0.05
HbA1c	NS	*P* < 0.05	NS	*P* < 0.05
Triglycerides	NS	NS	*P* < 0.05	*P* < 0.05

*P* < 0.05 means significant differences*; *NS: not significant.

**Table 4:** Healthy group compared with ketosis-onset and ketosis prone groups.

Group	Healthy group	Ketosis-onset group	Ketosis prone group
	(*n* = 50)	(*n* = 79)	(*n* = 80)
Gender (male : female)	13 : 12	51 : 28	11 : 9
Age (year)	38.37 ± 25.74	27.35 ± 20.82	44.85 ± 23.12
Age range (year)	12–65	7–55	22–72
BMI (kg/m^2^)	22.07 ± 5.23	22.18 ± 8.95	23.78 ± 9.27
FPG (mmol/L)	6.57 ± 1.12	16.75 ± 4.65	14.57 ± 3.23
HbA1c (%)	6.46 ± 0.93	11.45 ± 1.58	10.94 ± 2.08
Triglycerides (mmol/L)	1.24 ± 0.57	2.72 ± 2.63	2.72 ± 1.56
